# Synthetic Humic Acids Solubilize Otherwise Insoluble Phosphates to Improve Soil Fertility

**DOI:** 10.1002/anie.201911060

**Published:** 2019-12-04

**Authors:** Fan Yang, Shuaishuai Zhang, Jingpeng Song, Qing Du, Guixiang Li, Nadezda V. Tarakina, Markus Antonietti

**Affiliations:** ^1^ School of Water Conservancy and Civil Engineering Northeast Agricultural University Harbin 150030 China; ^2^ Max Planck Institute of Colloids and Interfaces Department of Colloid Chemistry 14476 Potsdam Germany

**Keywords:** humic acids, phosphorous, sustainable chemistry, plant fertilizers

## Abstract

Artificial humic acids (A‐HA) made from biomass in a hydrothermal process turn otherwise highly insoluble phosphates (e.g. iron phosphate as a model) into highly available phosphorus, which contributes to the fertility of soils and the coupled plant growth. A detailed electron microscopy study revealed etching of the primary iron phosphate crystals by the ‐COOH and phenolic groups of humic acids, but also illustrated the importance of the redox properties of humic matter on the nanoscale. The combined effects result in the formation of then bioavailable phosphate nanoparticles stabilized by humic matter. Typical agricultural chemical tests indicate that the content of total P and directly plant‐available P improved largely. Comparative pot planting experiments before and after treatment of phosphates with A‐HA demonstrate significantly enhanced plant growth, as quantified in higher aboveground and belowground plant biomass.

## Graphical abstract

**Making the P**: Artificial humic acids made from biomass in a hydrothermal process are a practical route to solubilize otherwise insoluble phosphorous species which contributes to the fertility of soils. Agricultural chemical tests indicate that the content of total P and directly plant‐available P improved.

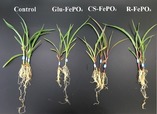

Phosphate fertilizers enable modern food production and are a dwindling resource.^1^ Available phosphorus (AP) is a main bottleneck which restricts crop growth in terrestrial ecosystems, and addition of phosphate fertilizers enhances primary food productivity^2^ and had set the base for the current population density. Globally, about 180 Mt are mined per year, and 50 % of the world's resources are mined in one country, Morocco, only. On the other hand, phosphates as such are very common, nearly omnipresent minerals, and there are around 50 to 1500 mg kg^−1^ of P in practically all soils,^3^ but most of these phosphates are not plant‐available as they show in fact too low solubility. Only a restricted class of mineral deposits is useful for fertilizer production, for example, by acid treatment and subsequent KOH neutralization, to set the base of the so called “KPN”, thus making “treatable phosphates” a rapidly shrinking resource. Traditional P fertilizer production is also accompanied by erosion and pollutant emissions.^4^ On top, most of the added fertilizer phosphate experiences rapid fixation in the soil, and only a small and variable fraction of the added P is directly available to the plants. Thus, an alternative, chemically targeted and controlled application of phosphate fertilizer involving a sustained enhancement of solubility and availability in soil is the key point to solve the phosphorus crisis, lastingly and world‐wide.^5^

This all is a well‐known problem, and many studies point out that soil organic matter has a positive effect on P solubilization and availability for plant growth, and even the activation of strongly mineralized phosphorus in the absence of fertilizers is described.^6^ Notably, plants often produce small amount of organic acid anions to solubilize P from soil in response to P deficiency.^3^ Organic acids, including humic and fulvic acids, can inhibit the precipitation of phosphate minerals, enhance the P bioavailability, and increase the availability of micronutrients.^7^ Organic acids have also been shown to induce high P mobilization,^8^ as they interact with multivalent metal cations and thus compete with PO_4_^3−^ for cation binding.^9^ Martinez et al.^10^ have suggested that organic matter, especially humic (HA) and fulvic (FA) acids, may dissolve insoluble phosphates, increasing phosphorus mobility by formation of humic acid‐metal‐phosphate complexes. The groups of Bolan^22^ and Oburger 8b indicated that soil treated with the trifunctional citric acid led to a larger increase in soluble P than that when treated with oxalic acid. Wang et al.^11^ reported that addition of humic acids to soil together with P fertilizer significantly increased the amount of directly available water soluble phosphate and strongly retarded the formation of mineralized phosphate: The P uptake and crop yield increased by 25 %, especially long after fertilization.

Solubilization of insoluble P into available P by soil organic matter is presumably a combination of physio‐chemical and biological mechanisms.6a The mechanism was assumed to be mostly related to the ability of organic acids (i.e., the total number of carboxyl and hydroxyl groups to complex metal cations and that way weakening the phosphate precipitation equilibrium. Many studies reported that plants have evolved several mechanisms to increase P bioavailability in the rhizosphere, of which, the exudation of low molecular‐weight organic acids is proposed as a key mechanism for increasing plant P uptake in soils. This is accompanied by the ability of some typical rhizobacteria to inhibit P back‐mineralization in the soil.^12^ From a physico‐chemical perspective, this main belief, however, looks too simple, and the involved processes are expected to be significantly more detailed and potentially even very different. This is why we analyze herein the chemical and structural details of phosphate transformation for a model, synthetic humic acid in a system free of biological contributions.

In our previous work, we described the generation of synthetic soil organic matter, including artificial fulvic and humic substances, by artificial hydrogeochemistry at higher temperatures and autogenous pressures in water.^13^ In the current work, we use these artificial humic acids for the reconstitution of insoluble phosphate minerals towards AP. The involved dissolution and complex mechanism are followed by advanced electron microscopy.

SEM images taken before and after the hydrothermal treatment already nicely illustrate that an insoluble phosphate rock minerals, in this case FePO_4_, and humic substances interact (Figure 1). The ability to “digest” minerals however exposes a massive diversity according to the biomass type the artificial humic acids were made of, which already contradicts the simplistic “acid” picture. Fulvic acids made of glucose as a carbohydrate model only etch and roughen the tip of the original FePO_4_ crystal (compare Figure 1 a and Figure 1 b), while humic acids derived from high lignin containing dried leaves results in a complete disintegration and restructuration of the crystalline bodies (Figure 1 c) into a “sponge”‐like structure.

**Figure 1:**
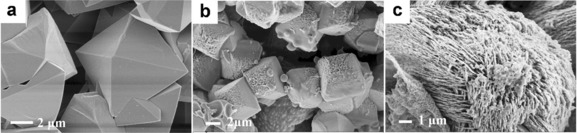
SEM images of original insoluble FePO_4_ (a), FePO_4_ reconstituted by fulvic acids from glucose (b), and FePO_4_ reconstituted by humic acids from leaves (c).

The corresponding SEM elemental mapping pictures (Supporting Information, Figure S1) reveal detailed information of etching mechanism. At the tip etched by fulvic acids, we find colocalization of carbon species, that is, strongly interacting acids drive by interfacial digestion and lowering of surface energies the dissolution of this Fe‐rich cutting plane even deep into the crystal.

With full biomass such as dried oak leaves to make the humic acid from, a different scenario sets in, and the whole big phosphate rock crystals turn into scaffolded lamellar superstructures with single nanolayer thicknesses of 30–50 nm. In addition, a surplus of humic matter is found as floccules attached onto the minerals. These humic acids prepared from leaf powders do not only have more acidic functional groups (Table S1), but also a high content of phenolic ‐OH which is heritage of the included lignin and its derivatives (>80 %). High‐resolution scanning transmission electron microscopy revealed the fine nanoscopic character and displays extended amounts of the amorphous phase, formed through etching with nanocrystalline particles embedded in it (Figure 2). Etching of crystal planes by biopolymers is a well‐known process and was described also by our groups.^14^ The presence of these nanoparticles indicates that the “sponge”‐like structures are not the more robust leftovers of an etching process, but rather the result of the formation of an intermediary amorphous phase which then recrystallizes via polyphasic nanoparticles:^15^ A similar precipitation of nanoparticles from an amorphous phase has been recently reported.^16^

**Figure 2:**
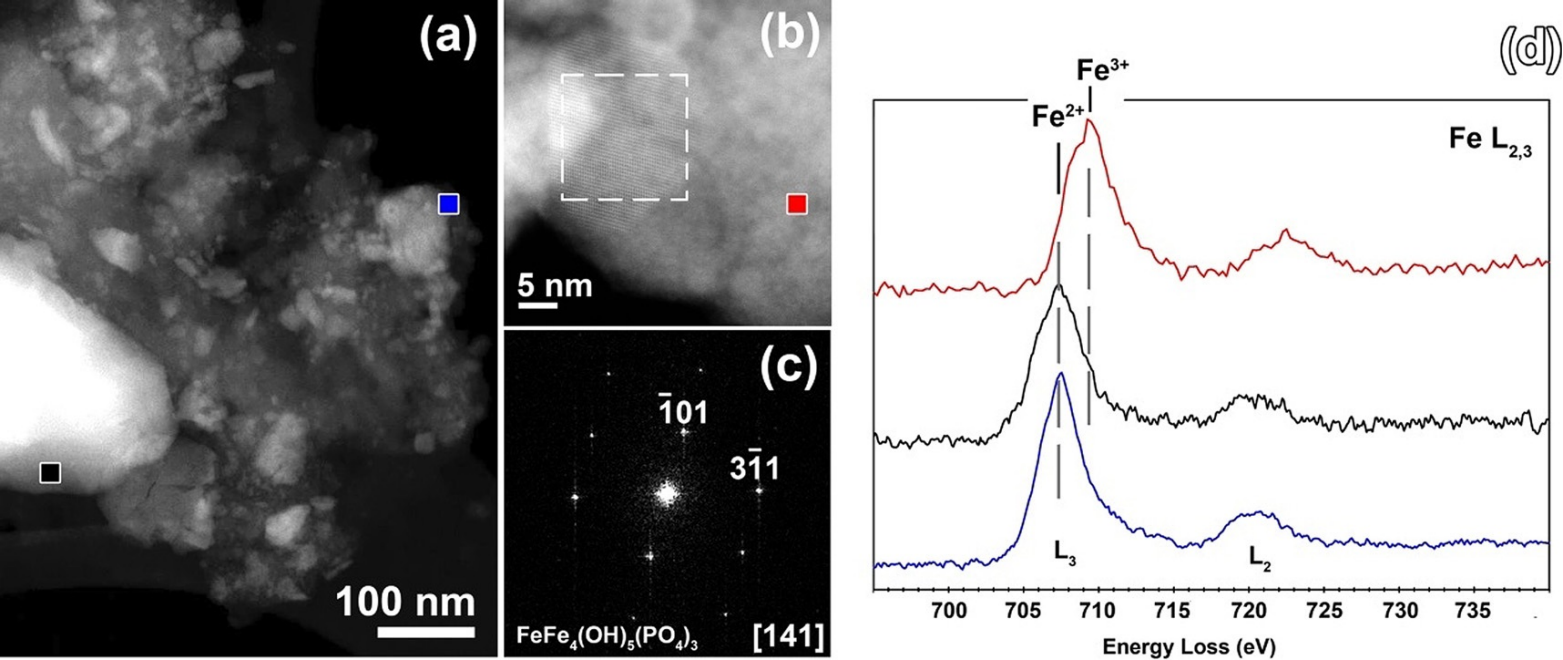
a) ADF‐STEM image of nanoparticles within the “sponge”‐like structure; b) HR ADF‐STEM image of the crystalline particle within the “sponge”‐like structure, the dotted line marks the area from which the Fast Fourier transformation, presented in (c), has been taken; d) Electron Energy Loss (EEL) spectra collected from positions in (a) and (b). The color of the marker corresponds to the color of the spectrum. The shift of the peak positions on EEL spectra indicate a reduction of the iron from Fe^3+^ to Fe^2+^, the extent of the reduction varies between different areas.

Analysis of EELS data and electron diffraction patterns obtained from nanoparticles clearly underline a parallel reduction of Fe from +3 to +2.^17^ Energy dispersive X‐ray spectroscopy maps (Supporting Information) show the presence of alkali metals in both nanoparticles and the amorphous phase. It is clear from those data that the dissolution of the Fe rock phosphate by leaf‐based A‐HA occurred by a redox process, most probably involving potassium ions for charge compensation. Indeed, the polyphenolic lignin is known to be highly redox active and can be even used in organic batteries^18^ and it is this redox strength which is obviously instrumentalized for an effective metamorphosis of the phosphate minerals.

This local picture is also confirmed by XPS and FTIR data (Figure S2, S3), indicating the strong chemical interactions between artificial humic matter and the creation of new mineral species, where morphological transformation is presumable thermodynamically driven by the large gain of interaction energy between the organic and the inorganic phase. This is then a pure physico‐chemical process driven by the interface activity of A‐HA. In order to verify the universality of our hydrothermal humic acid phosphorus solubilization, Apatite as an alternative phosphorus mineral was also treated. Figure S4 show again a complete change of morphology, together with a high C‐P colocalization for apatite (Table S2). Also, these structures look rather untypical for minerals, but are characteristic for mesocrystal formation from intermediate nanoparticles.

To approach to close‐to‐practical situation, we hydrothermally humified waste biomass together with a weak soil and added some insoluble iron phosphate crystals to perform hydrothermal humification (HTH) and phosphate activation in one shot to create artificial, fertilized soil. XRD analysis was carried out to determine the structure evolution during this process (Figure S5). The common diffraction peaks at 20.8°, 26.6°, 36.5°, 39.4°, 42.5°, 45.7° and 50.1° are typical for sandy, abundant minerals such as SiO_2_, Mg_2_SiO_4_, MgSiO_3_, Ca_3_Mg(SiO_4_)_2_ and some other oxides, meaning that the original soil composition was almost kept intact. The more relevant phosphate peaks match well with FePO_4_⋅2 H_2_O crystal plane (JCPDS No. 33‐0667) together with some tiny peaks implying impurities of iron oxyhydroxide (i.e., FeO(OH)). In some samples, we also find a few other peaks representing new P compounds which appeared, such as AlPO_4_, Mg(PO_3_)_2_, Fe(PO_3_)_3_, these are mostly porous phosphates and even activated metaphosphates. Obvious crystal transformations have also occurred in apatite‐based soil samples (Figure S6), that is, the original apatite crystals dissolved, and a new crystal structure of a hydrated (CaPO_3_(OH)⋅2 H_2_O) took its place, implying that apatite has experienced dissolution and recrystallization, in good agreement with SEM images (Figure S4). Besides, here some new soil‐mineral phosphate hybrid products can be found, such as Na_3_Mg(PO_4_)(CO_3_).

To base the planting experiments on these soils, the total P (TP) and available P (AP) was measured in natural and artificial high‐fertility soils according to standard soil tests (Figure S7 and Table S2). The TP content is in the typical range for natural fertile soils, namely, 965.9 P mg kg^−1^ for cultivatable soil (CS) and 900 P mg kg^−1^ for black soil (BS). The AP content is always much smaller (80.5 P mg kg^−1^ for CS and 180.0 P mg kg^−1^ for BS),^19^ which can be attribute to mineralization and re‐mineralization, which is less in the presence of humic matter.

The artificial LS‐FePO_4_ sample shows the highest AP values (2.14 and 14.3 times as much as that of black soil and sandy soil), coupled to the high soil organic matter (SOM) content (9.03 %), especially when compared to the original mineral “dirt” taken as a base for reinforcement (1.28 %; Table S2). The quantitatively demanded AP in farming soil is in range from 20–250 P mg kg^−1^. Generally, in black soil the organic P content accounts for 30–80 % of the total P pool,^20^ while most of the mineral P is not accessible or not available for plant roots.^21^ This is very different to the current case of artificial soils where—due to the hydrothermal preparation conditions—microbial life and the coupled organic P can be excluded, and all AP can be related to humic matter.

Comparative photographs of seedling growth process during 25‐day cultivation of corn seedlings in black soils (Figure 3 and Figure S8a) reveal that both aboveground and belowground plant biomass was significantly higher after treatment with FePO_4_‐based samples compared to control groups with only a 4‐day soil breaking time. The shoot‐to‐root ratios of weight were 3.18, 3.63, 3.11 and 2.32 in G‐FePO_4_, CS‐FePO_4_ and R‐FePO_4_ (G = Glucose, CS = corn stalk, R = old roots, i.e. the three biomasses which were used for A‐HA formation) treatment, and control, respectively. Significant increases in fresh and dry weight (Figure S8b,c) of both plant shoot and root biomass after application follow the improved plant nutrition. Moisture content also show a slight increase with highest values of 90.6 % in stem and 84.5 % in root among all groups (Figure S9), implying that the restructures phosphates allows accumulation of more nutrients and water in the early growth stage (25 days) of corn seedlings.

**Figure 3:**
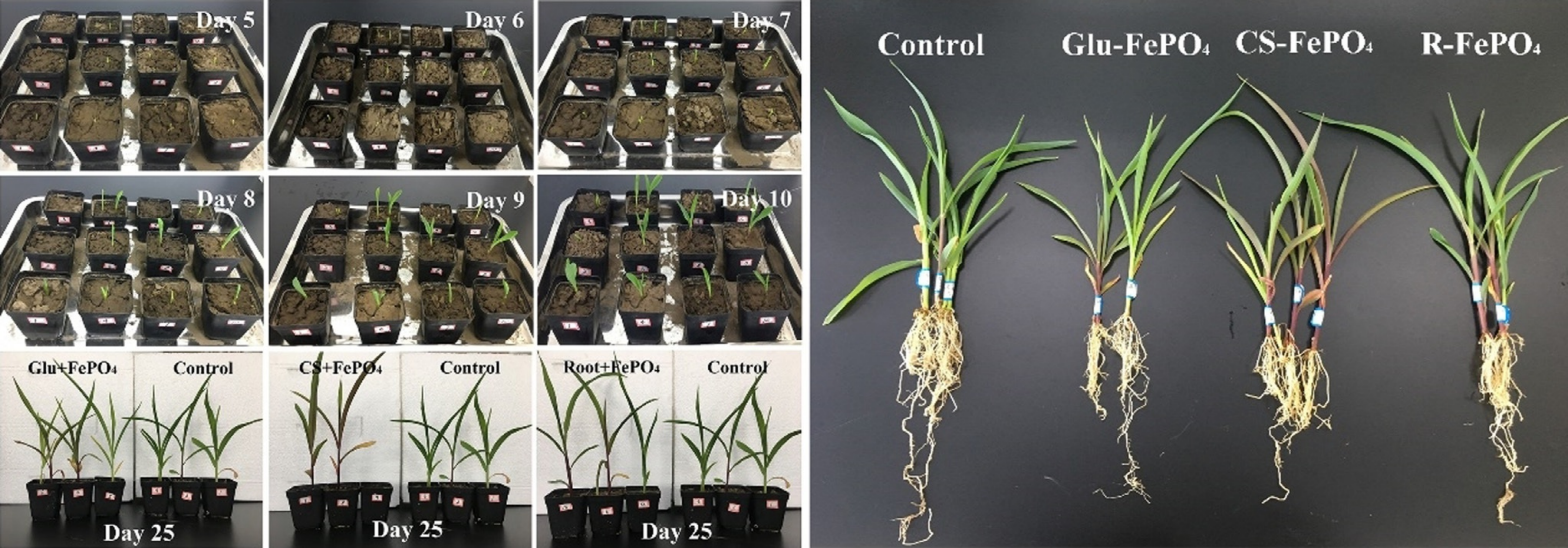
Comparative photos of seedling growth of corn seeds at 25 days in black soils with additions of P fertilizers derived from insoluble P rocks.

To conclude, we presented a practicable route for solubilization of otherwise insoluble phosphorous species by application of artificial humic substances using a “hydrothermal humification” technology. The metamorphosis was studied in model samples by a variety of analytical techniques, including HR(S)TEM and related spectroscopy techniques, which allowed to reveal not only the macroscopic but also the nanoscopic origin of this structural transformation. We found that the morphosynthetic changes are not only due to the classical mechanisms of organic acid etching, but also related to the polymer nature of humic acids and involved redox processes, all driven by artificial humic matter. The resulting “sponge”‐like morphological structures turned out to be humin‐stabilized, high surface area amorphous or polyphase mineral nanoparticle superstructures which do not undergo (at least immediate) remineralization. This colloidal dispersion of artificial humic matter and digested phosphate minerals was added to weak model soils, and planting experiments with corn seedlings indicated high levels of available P to promote plant growth, as confirmed by significantly higher daily nutrient uptake in control groups.

It is clear that any bigger claim, such as a possible contribution to future food safety, has to await more serious farmland testing, but also engineering optimizations. However, we consider the insights on how humic polymers can interfere with mineral surfaces, as well as the involved structure and crystal changes and the then enabled metabolization as highly promising to address this big question.

## Conflict of interest

The authors declare no conflict of interest.

## Supporting information

As a service to our authors and readers, this journal provides supporting information supplied by the authors. Such materials are peer reviewed and may be re‐organized for online delivery, but are not copy‐edited or typeset. Technical support issues arising from supporting information (other than missing files) should be addressed to the authors.

Supplementary
