# Supplementary material for: Synthetic Humic Acids Solubilize Otherwise Insoluble Phosphates to Improve Soil Fertility
Source: Angew Chem Int Ed Engl. 2019 Dec 4;58(52):18813–6. doi: 10.1002/anie.201911060 (PMC6973123; doi:10.1002/anie.201911060)
Supplement: Supplementary file 1 — Supplementary [file ANIE-58-18813-s001.pdf]

Angewandte Chemie
Eine Zeitschrift der Gesellschaft Deutscher Chemiker

Supporting Information

# Synthetic Humic Acids Solubilize Otherwise Insoluble Phosphates to Improve Soil Fertility

*Fan Yang,* Shuaishuai Zhang, Jingpeng Song, Qing Du, Guixiang Li, Nadezda V. Tarakina, and Markus Antonietti**

anie_201911060_sm_miscellaneous_information.pdf

# 1. Text

## SI-1. Materials and Methods

Unless otherwise noted, all of the commercial reagents were used as received. Glucose ($C_6H_{12}O_6$), iron phosphate ($FePO_4$), phosphorites/apatite ($Ca_5(PO_4)_3(OH)$) and potassium hydroxide (KOH) were purchased from Sigma-Aldrich Company. Various biomasses and sandy soils were collected in Brandenburg, Germany. Black soils for comparison were sampled in the campus of Northeast Agricultural University, Harbin, China. Before experiments, the crude biomasses are cut into a suitable size. Typically, 1.20 g rude biomass powders, 0.5 g insoluble P rock with various KOH qualities (ensuring the molar rations between degradable cellulose and alkali close to 1) in absence and presence of 5 g sandy soil and were put into the bottom of the glass tube in 50 ml autoclave in the oven to heat to 200 °C, and then keep for 24 h to insure the sufficient humification, then the products are collected after the temperature naturally cooled down to room temperature with subsequent drying procedure. In brief, the products are named as G-$FePO_4$ (glucose), L-$FePO_4$ (leaf), CS-$FePO_4$ (corn straw), R-$FePO_4$ (root) without the dirt addition and LS-$FePO_4$ and RS-$FePO_4$ with the dirt addition according.

## SI-2: SOM examination

Typically, SOM examinations of sandy soil, a series of artificial high-fertility soil and natural soils are carried out following the procedures: firstly, those empty porcelain boats were placed in a muffle furnace, burned at 95 °C for 30 min, taken out and cooled in a desiccator for 20 min, and the mass ($M_1$) was weighed; then, they were burned at the same temperature for 30 min, taken out, cooled, and weighed. Repeated the above steps, until their mass difference between the two times is less than 0.5 mg. Weigh out 0.50 g of dry sample in a known quality

porcelain boat, put it in an oven at 105 °C for 12h, then take it out, put it into the desiccator, cooled it, weighted it, recorded the mass ($M_3$); Then transfer these porcelain boats to a muffle furnace and heat up to 550 °C, burn for 5 h, cool in a desiccator, weigh, and record the mass ($M_2$) and calculate.

## SI-3: Soil phosphorus analysis

### 3.1 Total Phosphorus

Weigh out 0.25g of dried soil sample passing through 100-mesh sieve into 50 mL flask, moisten with a small amount of water, add 8 mL of concentrated sulfuric acid, shake and place overnight, then add perchloric acid 10 drips, shake well, set a small funnel at the mouth of the bottle, put it in an electric furnace (or heating device), heat it and dilute it until the solution in the bottle begins to turn white, continue to cook for 20 min, and all the cooking time is 45-60 min. The cooled digestion liquid is carefully washed into a 100 mL volumetric flask with water, and the water should be washed a small amount several times during rinsing. The flask was gently shaken, and after it was completely cooled, it was made up to volume with water, and the solution was filtered through a dry funnel and a non-phosphorus filter paper into a dry 100 mL flask. At the same time, it was a reagent blank test.

### 3.2 Soil available phosphorus

0.25 g of the soil sample, placed in a dry 150 mL Erlenmeyer flask, adding a leaching agent (c ($NaHCO_3$) = 0.5 mol $L^{-1}$, pH=8.5) 50.0 mL, it was stoppered with a rubber stopper and shaken on a reciprocating shaker at room temperature of (25 ± 1 °C) for 30 min.

## SI-4: Liquid phosphorus analysis

### 4.1 Total phosphorus

Procedure of acid peroxydisulphate digestion: transfer an aliquot of 50 mL filtrate into the 100 mL digestion volumetric flask. Add 0.6 g $K_2S_2O_8$ with a scoop and 3 mL 2 mol/L $H_2SO_4$ to the digestion flask and mix. Put flaks on the heat block 125°C for 90 min. Remove digestion flasks from the heat block and let it cool down to laboratory temperature. Make up to 100 mL with distilled water and mix. Filter through paper Filtrak, grade 389 or Whatman No. 40. The phosphorus concentration in soil water extracts was determined by means of the reduced phosphomolybdenum blue on a SKALAR auto-analyzer and by the ICP-AES technique on a Thermo Jarrell Ash Trace Scan analyser. The SKALAR automated procedure of phosphate determination is based on the following reaction: ammonium heptamolybdate and potassium antimony (III) oxide tartrate react in an acid medium with diluted solutions of phosphate to form an antimony-phosphomolybdate complex. This complex is reduced to an intensely blue-coloured complex by l(+)ascorbic acid. The complex is measured at 880 nm. The presence of P was analyzed with the molybdenum blue method, based on the reactions between P and $MoO_4$, Sb, and ascorbic acid. Total P concentration was determined using a colorimetric assay. Acid molybdate and Fiske's SubbaRow reducer solution were added to the digest to form a phospho-molybdenum complex. The total phosphorus (TP) was measured by calcination in a muffle furnace (550 °C) and was then extracted using a solution of 1 mol $L^{-1}$ HCl.

### 3.2 Available phosphorus

The P-solubility in neutral ammonium citrate ($P_{nac}$) is a widely accepted indicator for characterizing phosphate in hydrothermal products as a direct nutrient fertilizer for plant growth. The solubility of P was conducted to estimate the plant-availability of P in samples.

For $P_{nac}$, 500 μL of sample solutions were placed in 50 mL flask and extracted in 25.0 mL neutral ammonium citrate solution (1%) for 2 h at room temperature and stirring.

## 2. Figures

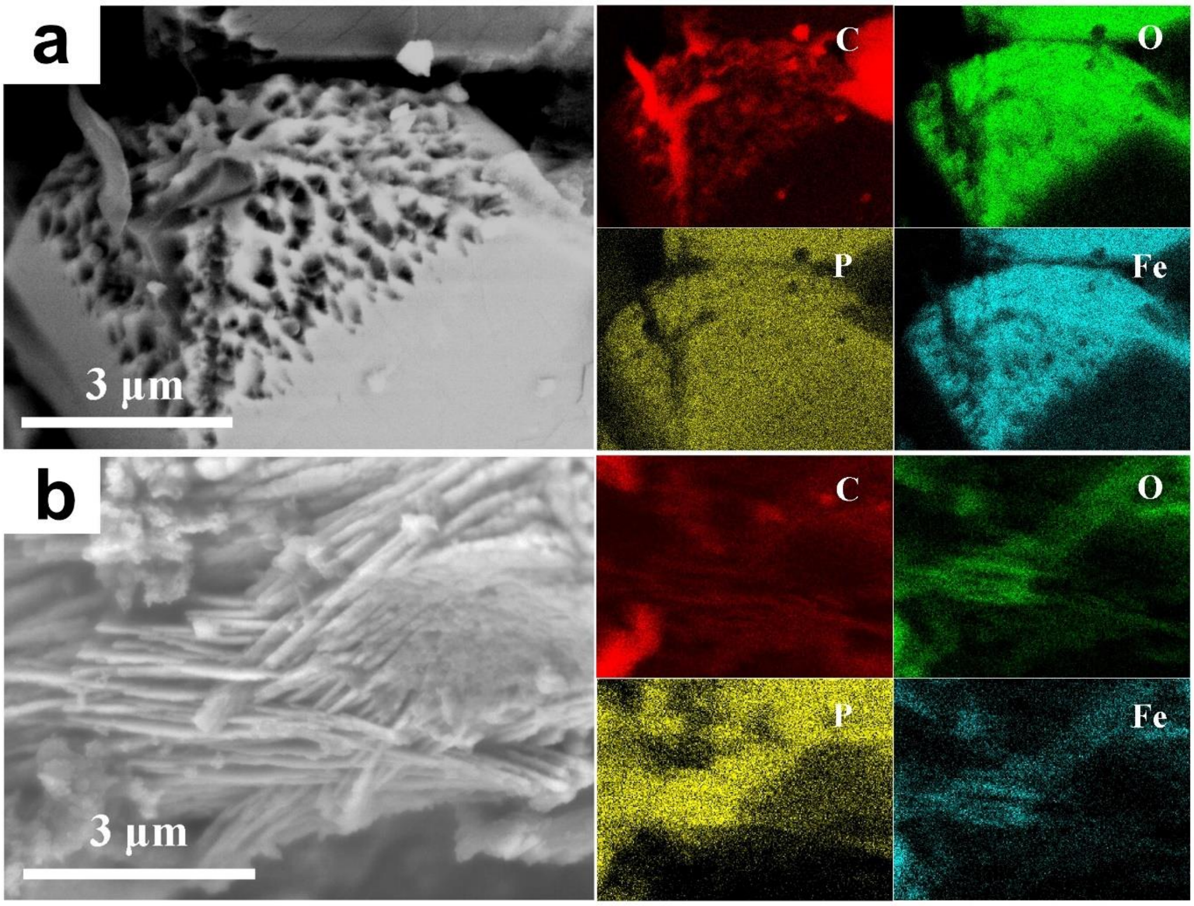


**Fig. S1.** Images and Corresponding elemental maps of insoluble P rock etched by humic substances prepared

with different biomass (a: glucose, b: leaf).

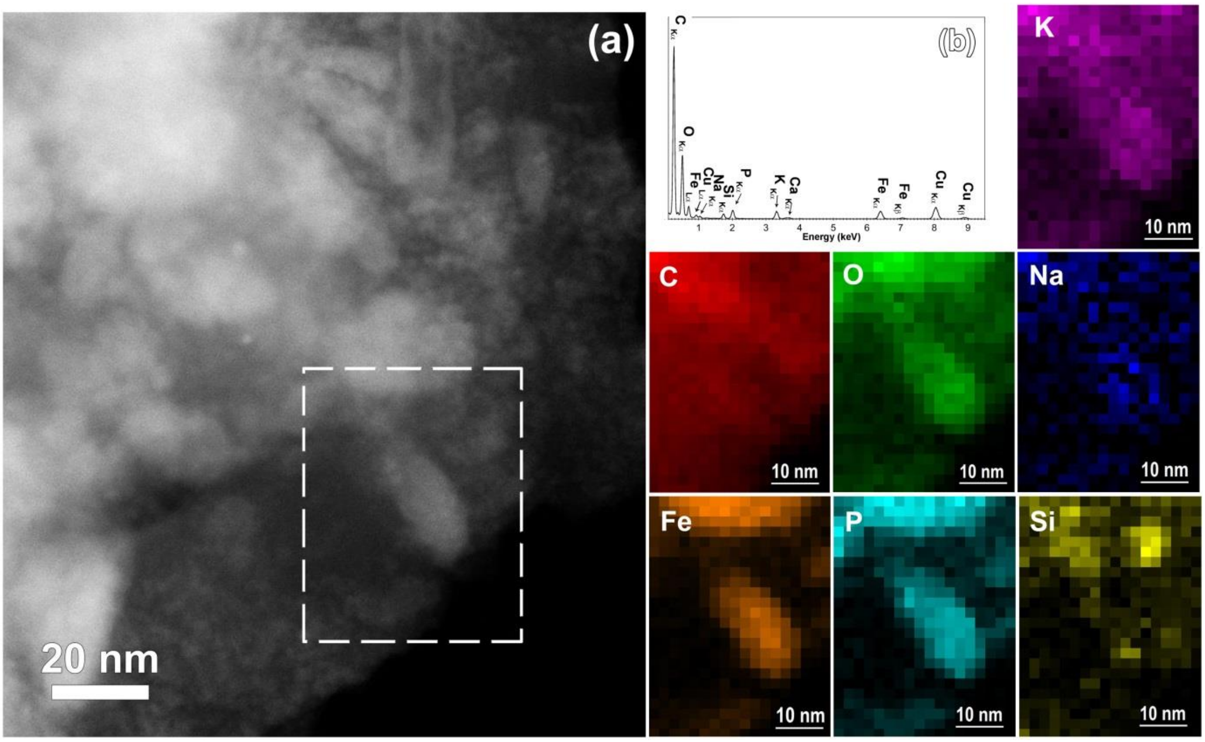


**Figure TEM-S:** (a) ADF-STEM image of nanoparticles within the "sponge"- like structure; (b) EDX maps representing signals of C, O, Na, K, Fe, P and Si, collected from the area marked by the dotted line in (a) and the overall EDX spectrum of this area.

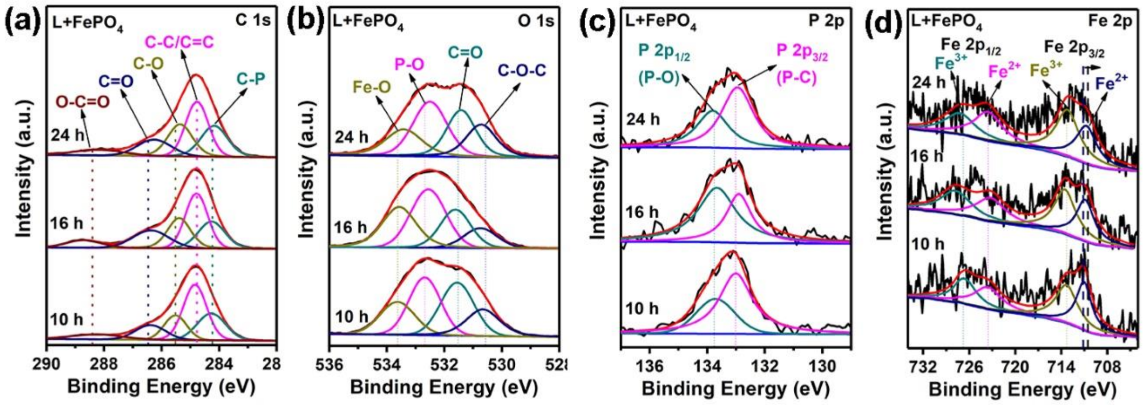


**Fig. S2.** C 1 s, O 1 s, P 2p and Fe 2p spectra of L-$FePO_4$ samples prepared at different time.

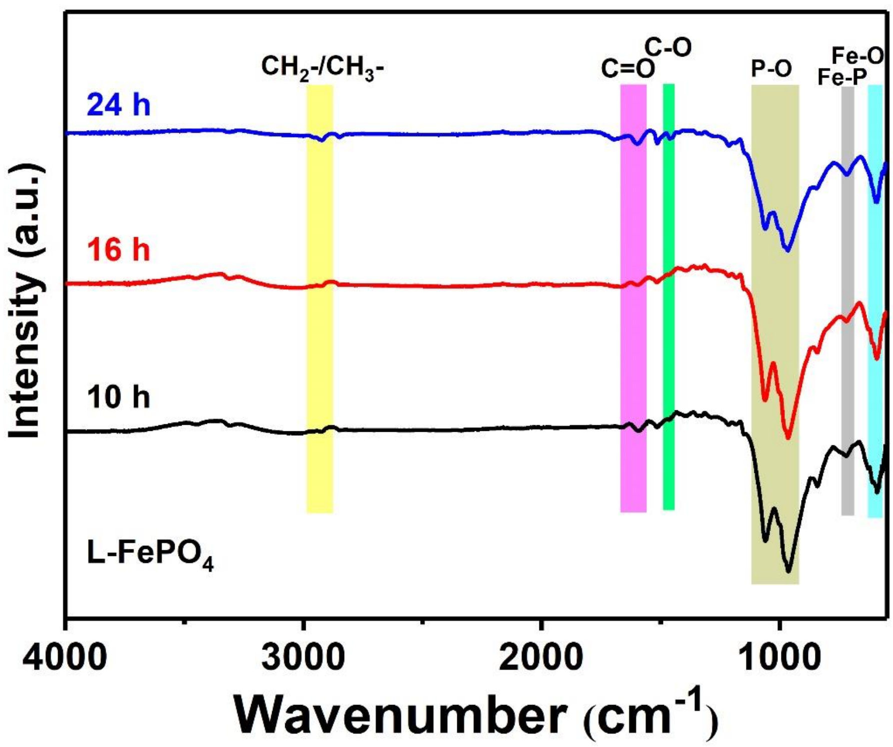


**Fig. S3.** FTIR spectrum of L-$FePO_4$ samples prepared at different time.

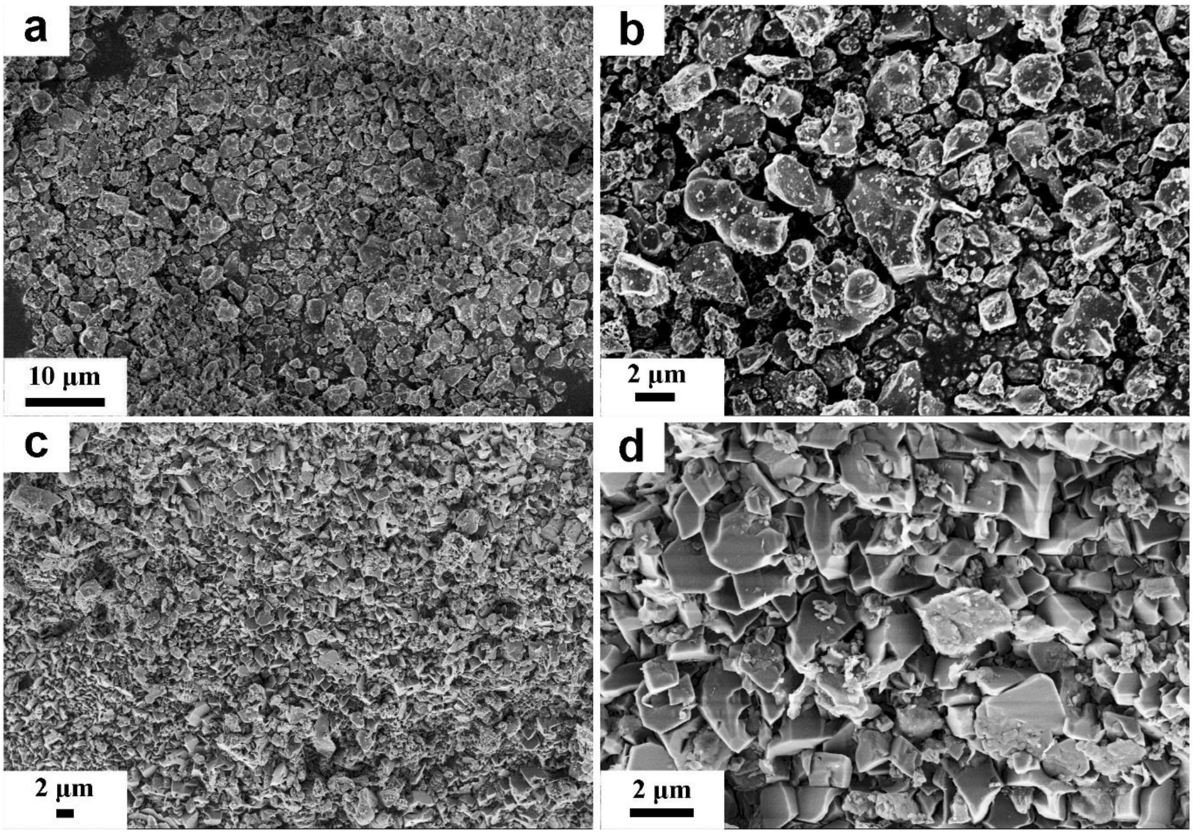


**Fig. S4.** SEM images for original apatite (a, b) and artificial apatite-based soil (c, d).

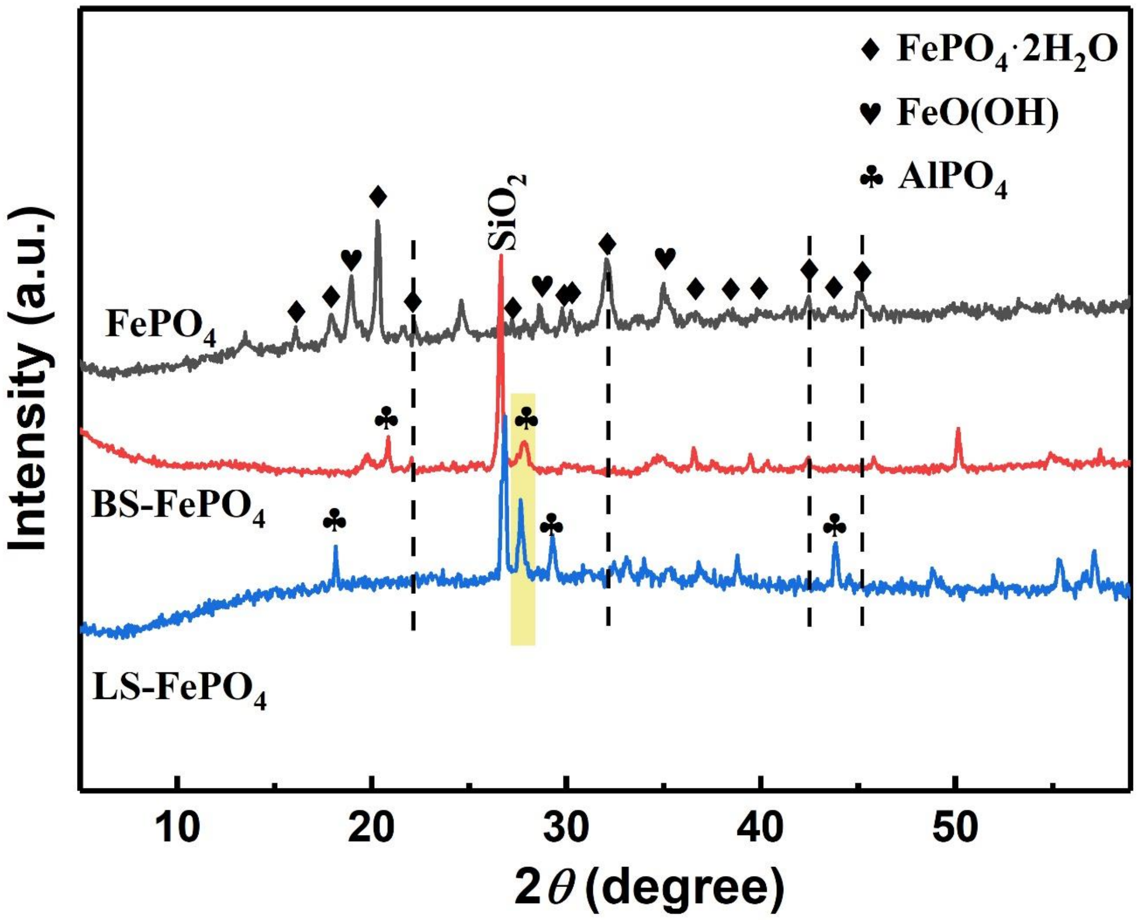


**Fig. S5.** Comparative XRD patterns for original $FePO_4$ rock and artificial high-fertility samples.

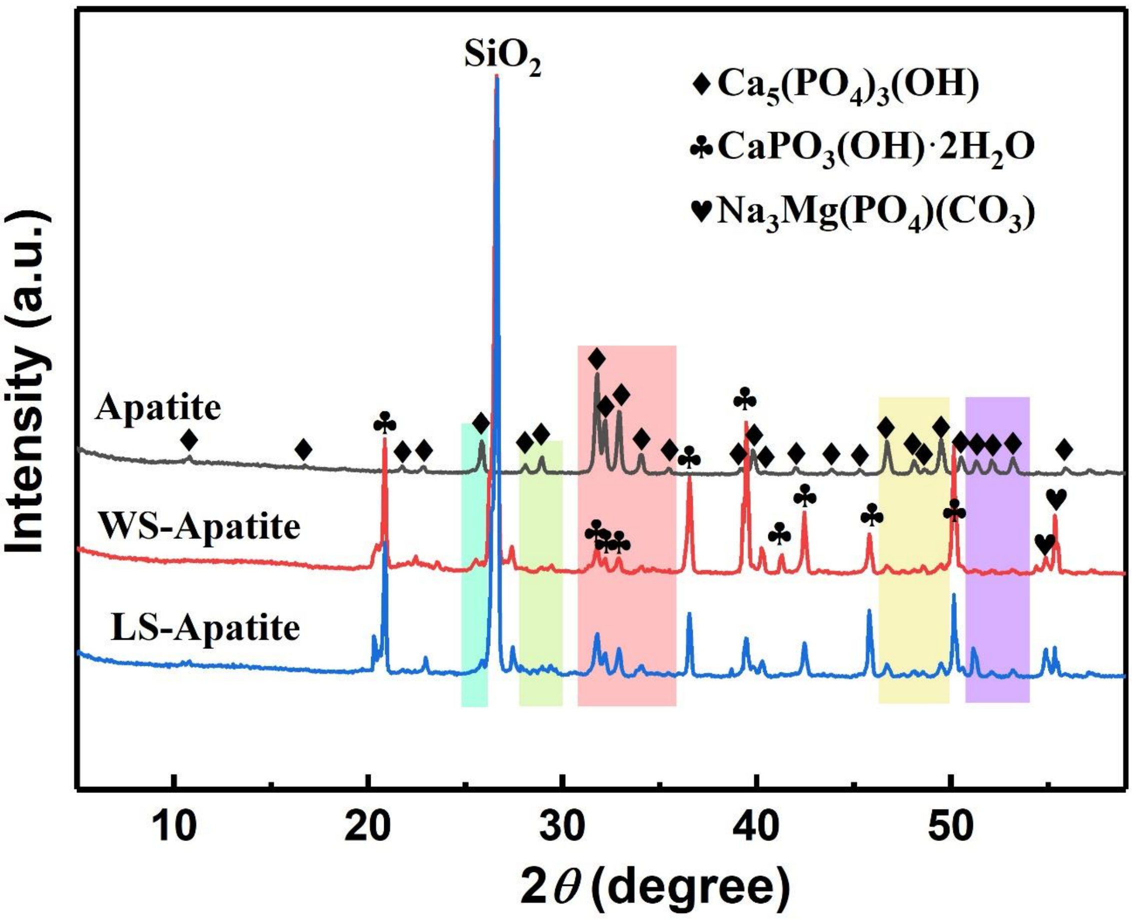


**Fig. S6.** Comparative XRD patterns for original apatite rock and artificial high-fertility samples.

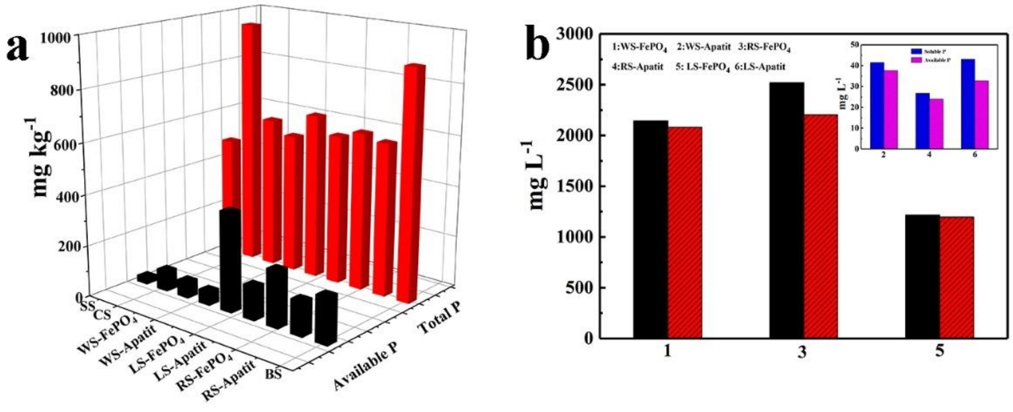


**Fig. S7.** Comparative results of available and soluble phosphorus in soil particles (a) and liquids (b) after artificial humification progress.

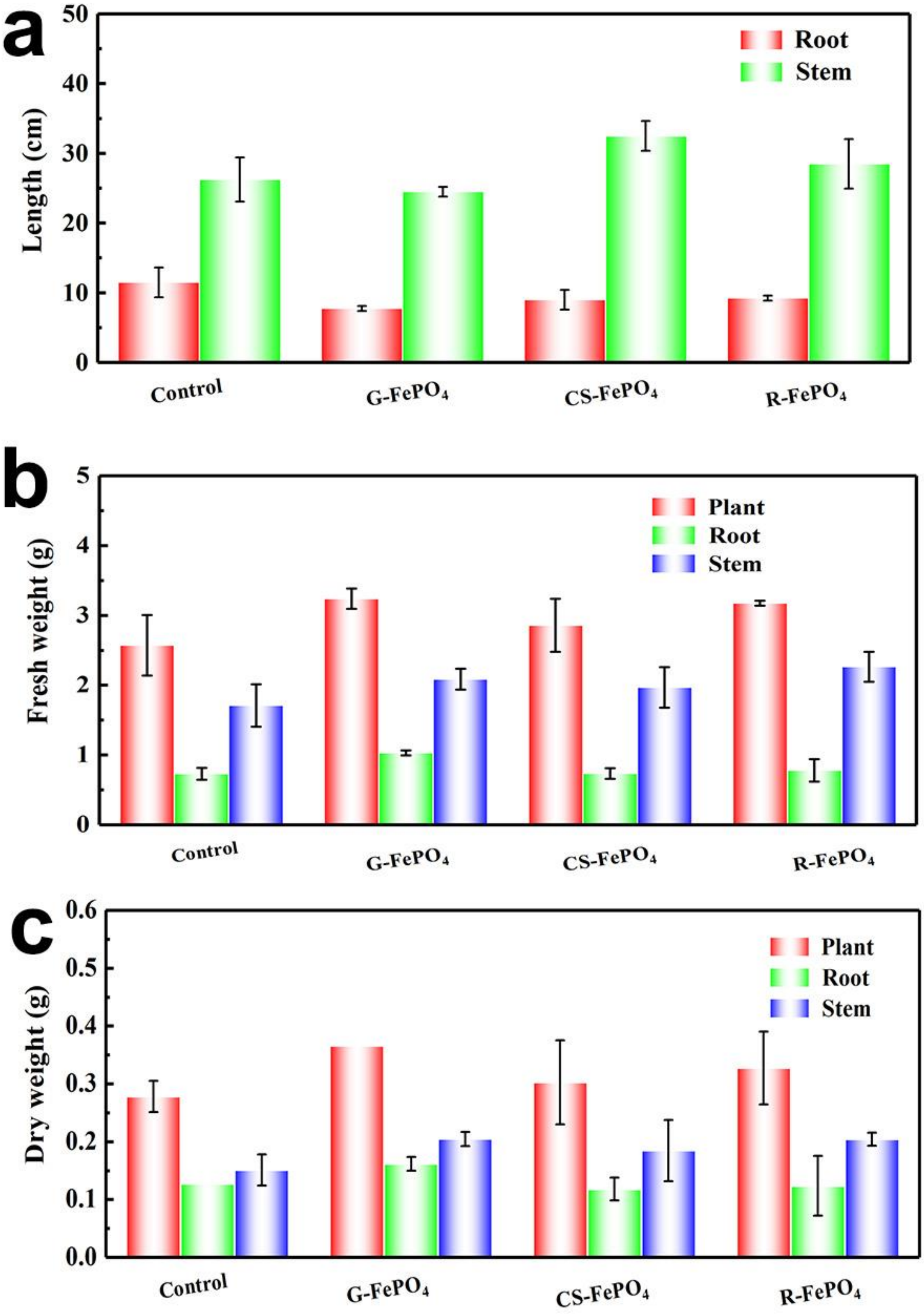


**Fig. S8.** Variations of length (a), (b) fresh weight and (c) dry weight of root, stem and plant after 25 days of growth in black soils with different additions.

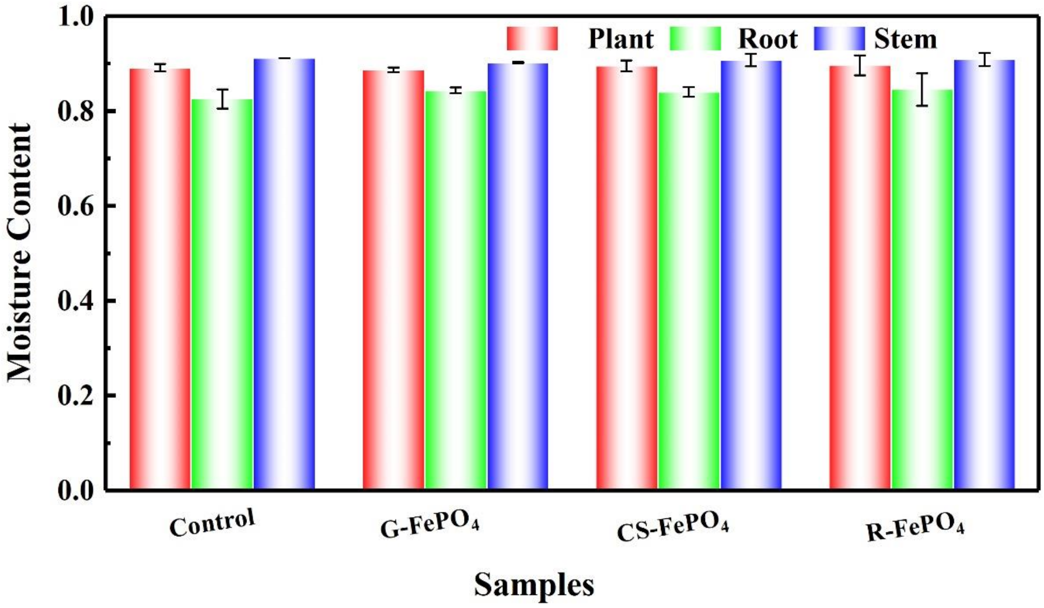


**Fig. S9.** Variations of moisture content after 25 days of growth in black soils with different additions.

## 3. Tables

**Table S1.** Surface acidic functional groups (mmol $L^{-1}$) and molecular weight of artificial humic substances.

| Samples | Phenolic | Carboxyl | Total acidity |
|---|---|---|---|
| **A-FA** | 1.88 | 1.62 | 3.50 |
| **A-HA[1]** | 8.34 | 1.66 | 10.00 |

**Table S2.** Comprehensive analysis of dissoluble P (DP), total P (TP) and available P (AP).

| Samples | $TP_{soil}$ (mg/kg) | $AP_{soil}$ (mg/kg) | $DP_{water}$ (mg/L) | $AP_{water}$ (mg/L) | SOM |
|---|---|---|---|---|---|
| **Sandy soil** | 474.9 | 27.0 | - | - | 1.28% |
| **Cultivated soil** | 965.9 | 80.5 | - | - | 8.83% |
| **WS-$FePO_4$** | 596.6 | 65.3 | 2143.8 | 2080.4 | 6.37% |
| **WS-Apatit** | 549.8 | 54.8 | 41.5 | 37.7 | 5.82% |
| **LS-$FePO_4$** | 649.4 | 386.5 | 1216.7 | 1196.1 | 9.03% |
| **LS-Apatit** | 587.4 | 131.9 | 43.0 | 32.7 | 8.89% |
| **RS-$FePO_4$** | 618.4 | 220.0 | 1127.3 | 994.5 | 12.38% |
| **RS-Apatit** | 598.7 | 135.0 | 31.8 | 27.8 | 10.12% |
| **Black soil** | 900.0 | 180.0 | - | - | 29.30% |

| | | | | | |
|---|---|---|---|---|---|
| **Grass soil** | 600.0 | 63.6 | - | - | 4.55% |

## Reference:


[1] Q. Du, G. Li, S. Zhang, J. Song, Y. zhao, F. Yang, 2020. High-dispersion zero-valent iron particles stabilized by artificial humic acid for lead ion removal. Journal of Hazardous Materials, 383, 121170.
